# Solid-State Fermentation of *Jatropha curcas* Cake by *Pleurotus ostreatus* or *Ganoderma lucidum* Mycelium to Determine Multi-Bioactivities

**DOI:** 10.3390/foods15020386

**Published:** 2026-01-21

**Authors:** Enrique Javier Olloqui, Emmanuel Pérez-Escalante, Raúl Velasco-Azorsa, Carlos Gutierrez, Juan Carlos Moreno-Seceña, Daniel Martínez-Carrera

**Affiliations:** 1 Centro de Biotecnología de Hongos Comestibles, Funcionales y Medicinales (CB-HCFM), Colegio de Postgraduados, Campus Puebla, Boulevard Forjadores de Puebla no. 205, Puebla 72760, Mexico; 2 Departamento deProducción Agrícola y Animal, Universidad Autónoma Metropolitana Unidad Xochimilco, Mexico City 04960, Mexico; 3 Área Académica de Química, Instituto de Ciencias Básicas e Ingeniería, Universidad Autónoma del Estado de Hidalgo, Carretera Pachuca-Tulancingo km 4.5, Mineral de la Reforma, Hidalgo 42185, Mexico; 4 Departamento de Biotecnología, División de Ciencias Biológicas y de la Salud, Universidad Autónoma Metropolitana, Campus Iztapalapa, Avenida San Rafael Atlixco 186, Mexico City 09340, Mexico; 5 Centro de Investigaciones Biológicas, Instituto de Ciencias Básicas e Ingeniería de la Universidad Autónoma del Estado de Hidalgo, Carretera Pachuca-Tulancingo km 4.5, Mineral de la Reforma, Hidalgo 42185, Mexico; 6 Departamento de Reproducción, Facultad de Medicina Veterinaria y Zootecnia, Universidad Nacional Autónoma de México, Mexico City 04510, Mexico; 7 Colegio de Postgraduados, Campus Montecillo, Carretera Mexico-Texcoco km 36.5, Texcoco 56264, Mexico

**Keywords:** *Jatropha curcas*, bioactive peptides, SSF, antioxidant, antidiabetic, antihypertensive, hypocholesterolemic, *Pleurotus ostreatus*, *Ganoderma lucidum*

## Abstract

Non-toxic *Jatropha curcas* cake is a by-product rich in protein that can be used in the food industry. Proteolytic kinetics were used to identify and quantify its antioxidant, antidiabetic, angiotensin-converting enzyme inhibitory, and hypocholesterolemic capacities. *J. curcas* cake was subjected to two systems of solid-state fermentation (SSF) hydrolysis by *Pleurotus ostreatus* (FPO) or *Ganoderma lucidum* (FGL), recording every 6 d until 24 d had passed. The maximum proteolytic capacity in FPO was reached on day 6 of the study, whereas FGL was achieved at 12 d. The FPO and FGL electrophoresis gels revealed the presence of 28 bands corresponding to peptides with molecular weights of less than 10 kDa in both systems analyzed. The highest FRAP values were obtained at 12 d for FPO and at the start of SSF for FGL. The highest antidiabetic capacity of FPO was obtained at 18 d and that of FGL at 24 d. The best antihypertensive activity obtained for FPO and FGL was observed at 6 d. FPO’s highest hypocholesterolemic activity was observed at the start of the SSF, while FGL’s was obtained at 24 d, which is the first report of the hypocholesterolemic activity of *J. curcas*. It should be noted that fermentation with *G. lucidum* outperformed fermentation with *P. ostreatus*, confirming its greater multi-bioactivity. The authors consider this method easy, practical, and generally recognized as safe (GRAS) for obtaining bioactive peptides.

## 1. Introduction

*Jatropha curcas* cake is a by-product of the biorefinery after oil extraction. According to nutritional research on non-toxic varieties, *Jatropha curcas* cake is distinguished by its high dietary protein content and favorable amino acid profile, highlighting its potential as a protein source in nutritional applications [1]. Given this high protein content, non-toxic *Jatropha* cake is emerging as a raw material for producing bioactive peptides, leveraging a biorefinery residue in the food industry [2].

Bioactive peptides (BPs) are molecules that usually contain 2–30 amino acids (<5 kDa) with biological activities that prevent various metabolic diseases with a wide range of activity and high biological specificity [3]. In addition, they do not bioaccumulate and are rapidly degraded. BPs are released from a matrix protein for activation, a process mediated by various hydrolysis mechanisms [4].

Enzymatic hydrolysis and microbial fermentation using bacteria, yeasts, and fungi are the most common hydrolysis methods used for producing BPs. In particular, fermentation processes are characterized by high levels of protease activity, low cost, and environmental friendliness [4]. In addition, fermentation is known to improve organoleptic, technological, and nutritional properties, such as food preservation and safety through bacteriocin production, improved texture and aroma generation, increased bacteriophage resistance, production of nutraceuticals such as oligosaccharides, reduction in toxic and anti-nutritional compounds, decreased biogenic amines, and release of BPs [5]. Bioactive peptides derived from microbial fermentation are generally considered safe, beneficial to health, and without adverse effects [6]. Fungal fermentation is generally performed using solid-state fermentation (SSF), a method that minimizes water usage, induces higher production of fungal protease, and simplifies product recovery compared to submerged fermentation [7]. Microscopic fungi of the genus *Aspergillus* spp., *Rhizopus* spp., *Flammulina* spp., *Actinomucor* spp., and yeast such as *Candida* spp. and *Saccharomyces* spp., among others, are used for these fermentations [8]. However, microscopic fungi may hydrolyze proteins indefinitely into amino acids for their own growth, thereby eliminating peptides with potential bioactive properties [9]. Recently, macrofungi have been used to ferment red bean flour, whole cow’s milk, and skim milk. Strains such as *Cordyceps militaris*, *Peniophora* sp., and *Neolentinus lepideus* were reported to produce antihypertensive peptides [10,11,12]. Therefore, research on fermentation using edible or medicinal fungi is increasingly important as an emerging method for obtaining bioactive peptides. Thus, the aim of the present study was to evaluate the hydrolysis kinetics of *J. curcas* cake by solid-state fermentation using *G. lucidum* and *P. ostreatus* and determine in vitro bioactivities, including antioxidant, antidiabetic, antihypertensive, and hypocholesterolemic capacities, as a practical, easy-to-use method for generating BPs.

## 2. Materials and Methods

### 2.1. Reagents, Materials, and Equipments

Tris-glycine system (SDS-Tris-Glycine-PAGE), 2,4,6-trinitrobenzene sulfonic acid (TNBS) solution at 5% (*w*/*v*), acetate buffer (CH_3_COONa anhydrous, ReagentPlus^®^ (≥99%), aqueous iron (III) chloride (FeCl_3_·6H_2_O ACS reagent (97%), iron (II) sulfate (FeSO_4_·7H_2_O, ReagentPlus^®^ (≥99%), 2,2-diphenyl-1-picrylhydrazil (DPPH), Trolox, dipeptidyl peptidase IV (DPP-IV), angiotensin-converting enzyme (ACE), hippuryl-histidyl-leucine substrate, borate buffer solution, pyridine (C_5_H_5_ N, anhydrous (99.8%), benzenesulfonyl chloride (99%), cholesterol, phosphatidylcholine, linoleic acid, sodium taurocholate and the cholesterol quantitation kit, (Cat. no. MAK043) were purchased from Sigma-Aldrich Chemicals Co. (Sigma-Aldrich, St. Louis, MO, USA). Glacial acetic acid (HPLC grade) and methanol (HPLC grade) were purchased from J.T. Baker (Center Valley, WA, USA). NaCl (ACS reagent), potassium carbonate solution (K_2_CO_3_ ACS reagent), and sodium phosphate buffer (NaH_2_PO_4_ and Na_2_HPO_4_ (ACS reagent)) were purchased from J.T. Baker (Phillipsburg, NJ, USA). 2,4,6-Tris(2-pyridyl)-s-triazine (TPTZ) (≥98%) and hydrochloric acid (dilution from commercial solution volumetric 0.1 M (0.1 N) HCl), endotoxin-free were purchased from Sigma-Aldrich/Merck KGaA (Darmstadt, Germany). Glycine (molecular biology grade) was purchased from Millipore Sigma (Burlington, MA, USA). Tris-HCl buffer (pH 8.0) was purchased from Thermo Fisher Scientific Inc. (Waltham, MA, USA). Nitrogen gas (Ultra High Purity (5.0)) was purchased from Infra^®^ (Guadalajara, Mexico). Microplate was purchased from Axygen (Union City, CA, USA). Bioactivity measurements were performed using a 96-well microplate reader (Epoch spectrophotometer, BioTek Instruments, Winooski, VT, USA). Acrylamide/bisacrylamide (37.5:1) stock solution with 2.7% cross-linker and Mini-Protean III gel electrophoresis equipment were purchased from Bio-Rad (Hercules, CA, USA).

### 2.2. Solid-State Fermentation of Jatropha curcas Cake

The non-toxic variety of *J. curcas* L. var. Sevangel cake devoid of phorbol esters was supplied by Centro de Desarrollo de Productos Bióticos (CEPROBI-IPN) in Morelos, Mexico. The oil was extracted mechanically at 7111.5 psi, 80 °C. The remaining cake was sun dried (24–32 °C) for 2 d on a dry, ventilated sieve and stored until analysis. This variety has been registered by national authorities (SIAP-SAGARPA, registration number: 1461), highlighting the undetectable values of phorbol esters by HPLC [1]. Percentage of each component in the proximate analysis of *J. curcas* var. Sevangel, according to official AOAC [13] methodologies, yielded the following results: 92.87 ± 0.05 dry matter (925.09B), 35.97 ± 1.17 crude protein (954.01), 12.80 ± 2.04 crude fat (920.39), ash 6.37 ± 0.09 (942.05), and crude fiber 41.92 ± 0.53 (985.29). The carbohydrate content (3.94%) was calculated by weight difference. The *J. curcas* cake was washed and soaked in distilled water at 21 °C for 1 h. After removing the water, the cake was autoclaved at 121 °C for 25 min and cooled to room temperature. The sample was weighed and placed in flasks and inoculated with fungi in aseptic conditions. *Pleurotus ostreatus* (CP-753) and *G. lucidum* (CP-145) were provided by the Center for Biotechnology of Edible, Functional, and Medicinal Fungi (CB-HCFM) in Puebla, Mexico. These edible and medicinal fungi were selected as good producers of proteases, and their broad adaptability across experimental conditions, including cultivation substrate, pH, and temperatures.

### 2.3. Fungal Hydrolysis

For each system inoculated with *P. ostreatus* (FPO) and *G. lucidum* (FGL), 2.5 mL of spore suspension per 100 g of wet *J. curcas* cake was added, and the mixture was incubated at 27 ± 3 °C for 24 d. A sample of *J. curcas* cake without inoculum was prepared as a control following the same procedure. Briefly, 125 mL of deionized water was added to each sample in triplicate at 0, 6, 12, 18, and 24 d at 130 rpm under oscillatory agitation to determine the degree of hydrolysis and the corresponding bioactivities. The proteolytic reaction was stopped by heating at 85 °C for 10 min. Finally, samples were centrifuged at 10,000 rpm for 10 min at 4 °C, and the supernatant was isolated and preserved at −18 °C for subsequent analysis.

### 2.4. Proteolytic Capacity

Proteolytic capacity was evaluated using the TNBS method. Peptides released during hydrolysis were separated by polyacrylamide gel electrophoresis (Tris-Glycine-SDS-PAGE).

#### 2.4.1. Free Amino Groups by the TNBS Method

The degree of hydrolysis was determined using the TNBS technique, following the methodology proposed by Le Maux et al. [14] with slight modifications. FPO and FGL samples (0.05 mL) were mixed with 0.05 mL of 0.21 M phosphate-buffered solution at pH 8.2 and 0.05 mL of 0.05% TNBS reagent (Sigma-Aldrich, St. Louis, MO, USA). The mixture was incubated at 50 °C for 60 min and protected from light. Subsequently, the reaction was stopped by adding 0.05 mL of 1 N HCl, and the absorbance was measured at 335 nm in a microplate reader. The concentration of free amino groups was quantified using a glycine calibration curve using with concentrations in the range of 0–200 ppm.

#### 2.4.2. Tris-Glycine Polyacrylamide Gel Electrophoresis (Tris-Glycine-SDS-PAGE)

Polyacrylamide gel electrophoresis was performed as described by Pérez-Escalante et al. [15]. A 15% separation gel and a 4% stacking gel were prepared from a 30% acrylamide/bisacrylamide (37.5:1) stock solution, using a 2.7% cross-linking agent. Image analysis was performed using Gel-Doc EZ software (Image Lab 6.1); Bio-Rad (Hercules, CA, USA).

### 2.5. Antioxidant Activity

#### 2.5.1. Ferric-Reducing Power (FRAP)

Antioxidant activity was evaluated as described by Pardo et al. [16]. A FRAP preparation from 300 mM acetate buffer at pH 3.6, 20 mM aqueous ferric chloride, and 10 mM TPTZ (prepared in 40 mM hydrochloric acid) in a 10:1:1 ratio. First, 20 µL of FPO or FGL samples and 280 µL of the FRAP preparation were added to each well. Absorbance was measured at 595 nm using a microplate reader. The results were compared with a calibration curve prepared with iron (II) sulfate, and the antioxidant activity was expressed in micromoles equivalent to iron (II) sulfate per 100 mL (mmol E Fe II/100 mL).

#### 2.5.2. Radical Scavenging Properties by DPPH

Radical scavenging capacity was determined as described by Brand-Williams et al. [17]. Briefly, a methanol solution of DPPH (0.1 mM) was prepared. Initially, 0.1 mL of FPO or FGL sample was combined with 2.9 mL of DPPH solution and incubated for 50 min protected from light. Absorbance was measured at 515 nm using a microplate reader and expressed in mmol of Trolox equivalent/100 mL (mg TE/100 mL).

### 2.6. Antidiabetic Capacity

The antidiabetic capacity was calculated using the (DPP-IV) inhibition assay described by Nongonierma and Fitzgerald [18]. Briefly, 25 µL of each FPO or FGL sample was added to a microplate well containing 50 µL of Gly-Pro-pNA reaction substrate (final concentration of 0.2 mM) in 0.1 M Tris-HCl buffer, pH 8.0. Next, 50 µL of DPP-IV diluted in the same buffer to a final concentration of 0.005 U/mL was added to each well. The mixture was incubated at 37 °C for 60 min. The reaction was stopped by adding 100 μL of 0.1 M potassium carbonate solution. Finally, the absorbance was determined at 405 nm using a microplate reader, and the percentage of DPP-IV inhibition was calculated using the following equation (Equation (1)):

(1) $$DPP\text{-}IV\;inhibition\;(\%)=\frac{A_{100}-(A_{S}-A_{SC})}{A_{100}}\times 100$$

where

*A*_100_ = absorbance at 405 nm of the enzyme reaction without hydrolysate.
*A_S_* = absorbance at 405 nm of the enzymatic reaction with hydrolysate.
*A_SC_* = absorbance at 405 nm of hydrolysate where enzyme and substrate were replaced by Tris-HCl buffer (pH 8) and potassium carbonate.

### 2.7. Antihypertensive Activity

Antihypertensive activity was assessed by measuring ACE inhibition, following the technique described by Cushman et al. [19], with some modifications.

A reaction mixture containing hippuryl-histidyl-leucine substrate (5 mM), borate buffer (0.1 M, pH 8.3), and NaCl (0.3 M) was prepared. Fifty microliters of the reaction mixture were added to 15 μL of the FPO or FGL sample. To initiate the reaction, 10 μL of ACE (0.1 U/mL) was added, and the mixture was incubated at 37 °C for 60 min. The reaction was stopped by adding 75 μL of 1.0 N HCl. Pyridine (150 μL) and benzenesulfonyl chloride (75 μL) were added to extract the formed hippuric acid, and the mixture was stirred and placed on ice. The mixture (200 μL) was placed in a microplate, and the absorbance was measured at 410 nm using a microplate reader. A blank (substrate + enzyme) and a control (substrate) were prepared. The following equation (Equation (2)) was used to calculate the percentage of inhibition:

(2) $$ACE\text{-}I\;inhibitory\;activity\;(\%)=\frac{A_{C}-A_{S}}{A_{C}-A_{B}}\times 100$$

where

*A_C_* = hippuric acid formed during the action of ACE without an inhibitor.
*A_B_* = unreacted hippuryl-histidyl-leucine that was extracted with ethyl acetate.
*A_S_* = hippuric acid formed after the action of ACE in the presence of an inhibitory substance.

### 2.8. Hypocholesterolemic Activity

An artificial micelle model was used to determine the hypocholesterolemic activity of the FPO and FGL samples, following the methodology described by Rendón-Rosales et al. [20], with slight modifications. For micelle formation, a lipid mixture containing phosphatidylcholine (2.4 mM) and linoleic acid (81 mM) was prepared, dissolved in methanol, and dried under nitrogen flow. The mixture was resuspended in phosphate buffer (15 mM, pH 7.2) containing sodium chloride (132 mM) and sodium taurocholate (6.6 mM), sonicated for 20 min, and incubated at 37 °C for 24 h. One milliliter of the micellar solution was mixed with 50 μL of the FPO or FGL sample, sonicated for 2 min, and incubated at 37 °C for 2 h. Finally, the samples were centrifuged, and the cholesterol content was quantified using a commercial colorimetric method according to the supplier’s instructions (Cholesterol Quantitation Kit). Cholestyramine was used as the positive control. The following equation (Equation (3)) was used to calculate the percentage of inhibition:

(3) $$Cholesterol\;inhibitory\;(\%)=\frac{Ch_{0}-{Ch}_{S}}{{Ch}_{0}}\times 100$$

where

*Ch*_0_ = Cholesterol content of micelles.
*Ch_S_* = cholesterol content remaining in the micelles with the sample.

### 2.9. Statistical Analysis

All experiments were performed in triplicate. Data are presented as mean ± standard deviation and were analyzed using one-way ANOVA. Significant differences were determined using Tukey’s post hoc test with a significance level of 0.05. Statistical analyses were performed using the Statgraphics Centurion XVI.I software.

## 3. Results and Discussion

### 3.1. Proteolytic Capacity

#### 3.1.1. Analysis of Free Amino Groups

As shown in Table 1 the free amino groups increased for both SSF systems during the fungal hydrolysis. A similar increase (*p* ≤ 0.05) was observed in the FPO system at 6 and 18 d (2014.23 ± 26.86 and 2060.85 ± 40.84 ppm). After this increase, a decrease (*p* ≤ 0.05) occurred at 12 and 24 d (1679.34 ± 44.19 and 1828.56 ± 22.74 ppm), respectively. *P. ostreatus* exhibits wide variability in peptidases with specific cleavage sites, explaining the initial increase on day 6. By contrast, the decrease on day 12 may be the result of interactions between peptides (formation of aggregates, precipitates, and steric hindrance) that inhibit enzymatic activity and the interaction of amino groups with the TNBS reagent [21,22,23]. As hydrolysis progressed, the enzymes continued to cleave the protein substrate and release new amino groups for reaction with TNBS, resulting in a detectable increase on day 18 and lower activity on day 24. These fluctuations in amino group concentrations are similar to those reported for the Flavourzyme enzyme (a mixture of endopeptidases and exopeptidases) in amaranth seed hydrolysis systems [24]. In the FGL system, an increase was observed at 12 d, reaching 1912.67 ± 72.70 ppm, with no statistical difference in subsequent measurements (*p* ≤ 0.05). Consequently, the FGL system exhibited a steady increase in the degree of hydrolysis throughout the hydrolysis period. The decrease in proteolytic activity in the FPO system may be due to *P. ostreatus* protease inhibitors synthesized from the substrate, such as aspartic peptidases, cysteine peptidases, serine peptidases, and metallopeptidases [25]. Even temperatures below 22–30 °C can decrease protease activity [26]. Laccases are enzymes produced by *P. ostreatus* that are involved in lignin degradation. An inverse relationship has been observed between laccase and protease activities [27], and laccase activity may decrease proteolytic activity.

#### 3.1.2. Separation of Peptides by Tris-Glycine-SDS-PAGE

**Figure 1:**
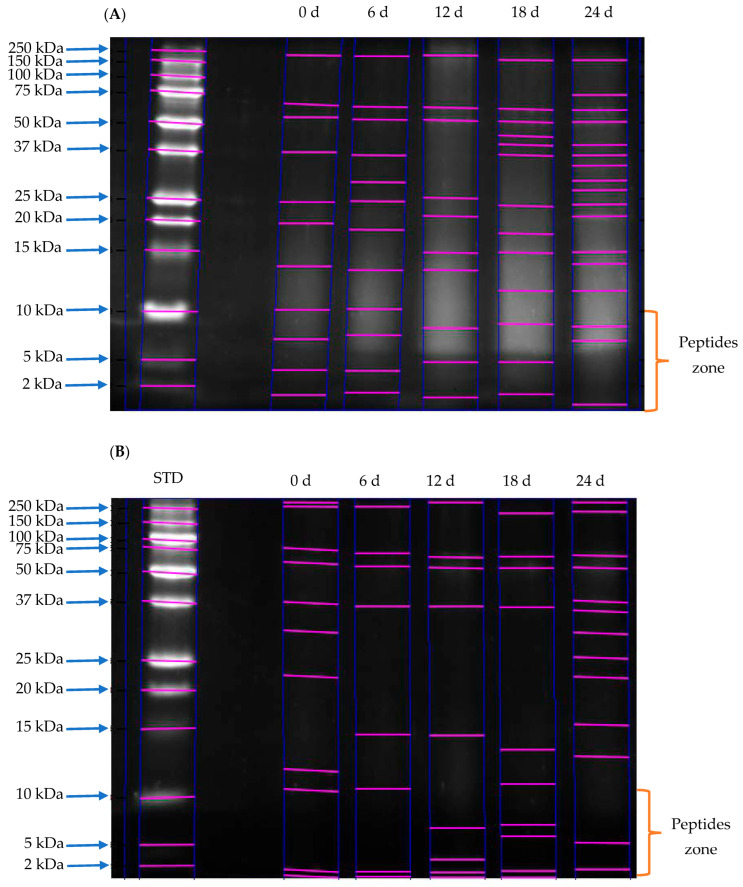
Peptide separation by Tris-Glycine-SDS-PAGE in *J. curcas* cake hydrolyzed with *P. ostreatus* (**A**) and hydrolyzed *Jatropha* cake with *G. lucidum* (**B**). STD: peptide standard. Hydrolysis time (0–24 d). The molecular mass range of peptides from 2 to 250 kDa is depicted on the left side of panels (**A**,**B**). The pink lines represent the bands identified in each lane of the electrophoresis gel. The peptides of particular interest in this study were located within the 2–10 kDa range.

Gel electrophoresis analysis revealed that the cake from *Jatropha curcas* L. var. Sevangel exhibited a protein profile with 11 bands ranging from 2 to 250 kDa (Figure 1). At 12 days, both macrofungal hydrolysis systems showed an increase in the number of low-molecular-weight bands, indicating enhanced production of smaller peptides. At 0 and 6 d, a low number of electrophoretic bands was observed due to a low degree of hydrolysis (Table 1). Both systems efficiently and viably hydrolyzed the protein hydrolysis of non-toxic *J. curcas* cake to produce smaller peptides.

*J. curcas* L. var. Sevangel cake contains globulins and glutelins with weights of 15–50 kDa [28], with degradation observed as the hydrolysis time increased by proteolytic enzymes from the mycelium of *P. ostreatus* and *G. lucidum*. Peptides obtained from *J. curcas* seeds exhibit immunomodulatory, antimicrobial, and antimalarial bioactivities, with molecular weights ranging from 693 to 1342 Da [29]. In addition to these bioactivities, there is evidence of peptides with antioxidant, antihypertensive, antidiabetic, and hypocholesterolemic properties that are smaller than 10 kDa [4]. Gel electrophoresis of FPO and FGL samples revealed peptide bands corresponding to molecular weights below 10 kDa in both systems analyzed, indicating the presence of bioactive peptides.

More peptides ≤ 10 kDa were found in the FGL system than in the FPO system at 12 and 18 days (Figure 1). This finding is consistent with the greater hydrolytic capacity of the FGL system relative to FPO system. The increase in low-molecular-weight peptides reported in this study was lower than that reported in a study on a non-toxic *J. curcas* protein isolate, which was enzymatically hydrolyzed with Alcalase, and was similar to that reported with Flavourzyme [2]. Unlike protein isolates, *J. curcas* cake contains 41.92 ± 0.53% fiber, which promotes mycelium growth during optimal solid-state fermentation [30]. A disadvantage of enzymatic hydrolysis is the high cost of enzymes and low yields [4]. Unlike other enzymatic hydrolysis methods, the macrofungal hydrolysis in this study stands out for being a simple and economical process for peptide hydrolysis in *J. curcas* L. var. Sevangel.

**Figure 2:**
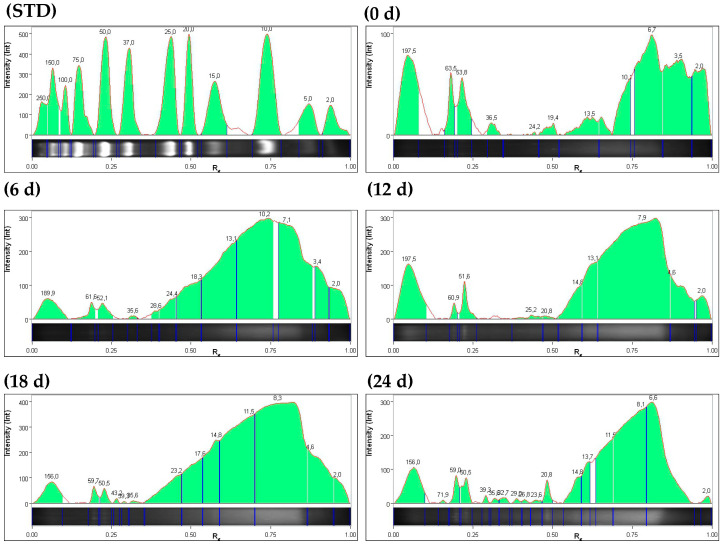
Electropherogram profiles at 0, 6, 12, 18, and 24 h in *J. curcas* cake hydrolysate with *P. ostreatus*. The green-shaded region in each figure corresponds to the peptide molecular weight concentrations. The data indicated an increase in the concentrations of multiple peptides with molecular weights ≤ 10 kDa relative to the initial time point.

**Figure 3:**
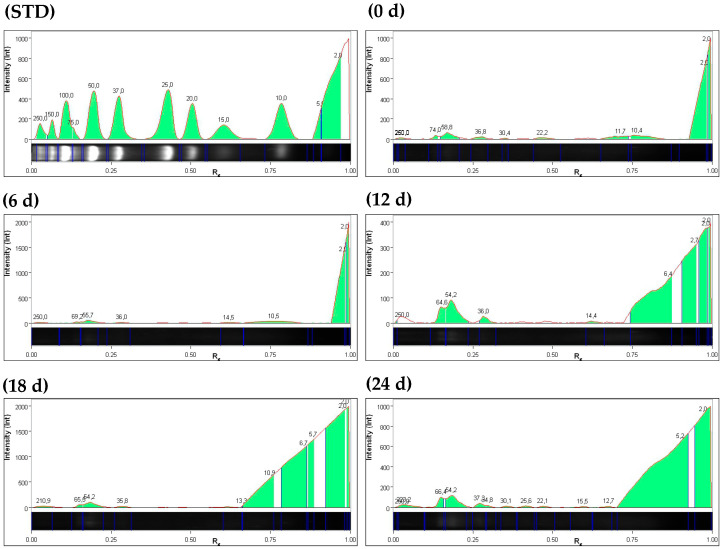
Electropherogram profiles at 0, 6, 12, 18, and 24 d in *J. curcas* cake hydrolysate with *G. lucidum*. The green area in each figure represents the peptide molecular weight concentration. This figure shows an increase in the concentrations of several peptides ≤ 10 kDa from 12 d.

In both hydrolysis systems, an increase in hydrolysis was observed from the initial time of study; peptides reported ≤ 10 kDa at initial time (0), 6, 12, 18, and 24 d for the FPO system were 55.2, 32.8, 71.1, 52.3, and 61.1%, respectively. In the FGL system, 72.2, 80.2, 70.4, 66, and 83% of peptides were reported ≤ 10 kDa at 0, 6, 12, 18, and 24 d, respectively (Figure 2 and Figure 3, Supplementary Materials). No relationship was observed between time 12 d (higher peptide concentration of ≤10 kDa) in the FPO system and the highest values of reported bioactivities. In contrast, in the FGL system, the reductive power of FRAP, DPP-IV inhibition, and hypocholesterolemic activity were highest at 24 days, coinciding with a higher concentration of peptides ≤ 10 kDa (Table 1).

### 3.2. Antioxidant Activity

Antioxidant activity was evaluated using two methods: the FRAP test, which measures reducing power, and the DPPH test, which measures free radical scavenging activity in both *J. curcas* cake hydrolysis systems.

**Figure 4:**
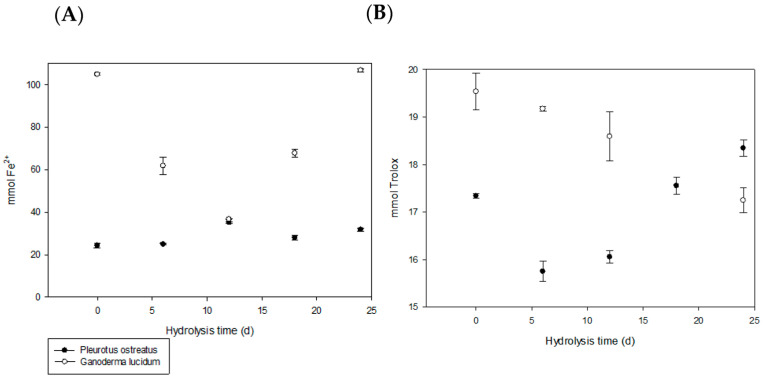
Antioxidant activities measured by FRAP (**A**) and DPPH (**B**) assays using *Pleurotus ostreatus* and *Ganoderma lucidum* derived from *Jatropha curcas* L. var. Sevangel cake. Results are presented as mean ± standard error per 100 mL. FRAP refers to ferric-reducing antioxidant power, and DPPH to 2,2-diphenyl-1-picrylhydrazyl radical scavenging activity; hydrolysis times ranged from 0 to 24 d. The data indicate that the *Ganoderma lucidum* system (FGL) exhibited greater reducing power than the *Pleurotus ostreatus* system (FPO) at days 0, 6, 18, and 24. Regarding free radical scavenging activity, the FGL system demonstrated superior performance during the initial hydrolysis period (0–12 d).

The results are presented in Table 1 and Figure 4, which show the antioxidant properties observed at the control times. In the FRAP test, a linear increase occurred up to 12 d of hydrolysis in the FPO system (*p* ≤ 0.05). On the other hand, the FGL system exhibited the highest reductive activity at the start of hydrolysis and after 24 d of hydrolysis. The reductive activity at the start of hydrolysis is due to the antioxidant capacity of *G. lucidum*. The antioxidant activity of *G. lucidum* mainly derives from glycerides, many of which are hydrophilic [31], and whose extraction increases at high temperatures (≥106.45 °C) [32]. In addition, ganoderic acids (bioactive triterpenoids present in *G. lucidum*) provide a defense mechanism against reactive oxygen species; however, ganoderic acids degrade rapidly at temperatures above 75 °C for 2 h [33].

In contrast, the antioxidant peptides in *G. lucidum* exhibit more potent free-radical scavenging activity [34]. In a recent study, antioxidant peptides were identified in *G. lucidum* using a machine learning-based evaluation strategy [35]. Therefore, the bioactive compounds present in *G. lucidum* have a high reductive capacity, and the FGL system in non-toxic *J. curcas* increases its reductive activity again after 24 d of hydrolysis without showing any differences compared to the start of hydrolysis.

### 3.3. Antidiabetic Capacity

The ability of peptides generated by hydrolysis with *P. ostreatus* and *G. lucidum* to inhibit DPP-IV was evaluated (Table 1). At the onset of hydrolysis, the observed values were 5.87% and 9.79% for FPO and FGL, respectively. In the FPO system, the maximum value was observed at 18 d, whereas in the FGL system, it was observed at 24 d, with 46.12% and 86.43%, respectively (*p* ≤ 0.05).

*J. curcas* has been the subject of antidiabetic studies, as drinking tea from it is associated with this effect. Tea is primarily consumed as a beverage made from leaves, fruits, or bark extracts for diabetes management [36]. Proteases with antidiabetic activity have also been reported in the cake of toxic *J. curcas* varieties [28].

In a previous study, enzymatic hydrolysis was performed with Alcalase and Flavourzyme on non-toxic *J. curcas* cake, reaching maximum values of 68.86% and 52.75%, respectively; in both systems, the highest values were obtained after 6 h of hydrolysis [2]. In this study, particularly in the FGL system, 86.43% DPP-IV inhibition was achieved at 24 d, exceeding the values obtained with Alcalase. This can be attributed to the proteolytic activity of *G. lucidum,* which cleaves specific sites of the parental protein in *J. curcas* cake. Further experiments are needed to elucidate the proteolytic mechanisms of *G. lucidum*. This method could be considered viable, efficient, eco-friendly, and safe for obtaining peptides with antidiabetic activity from non-toxic *J. curcas* cake.

The FGL system showed results similar to those previously reported for protein isolates from beans subjected to enzymatic hydrolysis, where the fraction <1 kDa reached 55.3% [37] and 96.7% DPP-IV inhibition [38].

### 3.4. Antihypertensive Activity

Table 1 presents the inhibitory activity results obtained from both hydrolysis systems. At the start of hydrolysis, the FPO and FGL systems exhibited ACE-I inhibition percentages of 51.25% and 59.68%, respectively. These values indicate that *J. curcas* contains antihypertensive compounds in its own matrix. Similar results were observed in enzymatic hydrolysates (Alcalase and Flavourzyme) of *J. curcas* L. var. Sevangel [2]. Both systems showed increased ACE-I inhibitory activity from 6 d, with values of 61.76% and 66.49%, respectively (*p* ≤ 0.05).

The ACE-I inhibitory activity has been studied in hydrolysis systems carried out by basidiomycete fungi. Xiao et al. [10] conducted solid-state fermentation of *C. militaris* in red bean flour for 7 days, thereby improving physicochemical and functional properties and demonstrating ACE-inhibitory activity.

On the other hand, Okamoto et al. [11] carried out submerged fermentation of milk using mycelium of the brown rot fungus *Neolentinus lepideus*, achieving values of 80% ACE inhibition. This study highlights the production of bioactive peptides by basidiomycete fungi as a viable and cost-effective mass-production system.

Similarly, Okamoto et al. [12] performed submerged fermentation of cow’s milk with the mycelium of the white rot fungus *Peniophora* sp. The basidiomycete used lactose as a substrate and produced Tyr-Pro, Phe-Pro, and Val-Pro peptides, which have been reported to show ACE-inhibitory activity. This hydrolysis system was suitable for the release of antihypertensive peptides with ACE inhibition of 60–70% after 4–11 d of fermentation. These results are similar to the maximum values obtained after 6 d of fermentation in both hydrolysis systems.

These studies highlight the production of bioactive peptides from diverse substrates via hydrolysis of basidiomycete mycelium as a cost-effective, sustainable, and efficient method.

### 3.5. Hypocholesterolemic Activity

Hypocholesterolemic activity was achieved by suppressing the micellar solubility of cholesterol, which may be similar to the decrease in serum cholesterol [39]. The highest percentage of hypocholesterolemic activity in FPO was at the beginning of hydrolysis, with 56.48% (Table 1). *P. ostreatus* has been previously reported to have a hypocholesterolemic effect [40,41]. These anti-atherogenic properties likely involve hydrophilic activity and can be observed at the onset of FPO hydrolysis in the study. Subsequently, they declined over the course of the study.

Likewise, aqueous extracts of *G. lucidum* have cholesterol-lowering effects [42]. For this reason, a high percentage of hypocholesterolemic activity can be observed at the beginning of hydrolysis. Consequently, a decrease and a maximum increase are observed at 24 d of hydrolysis (Table 1). It has been observed that small peptides have greater hypocholesterolemic activity than larger ones (0–5 vs. 5–30 kDa) [43]. This argument aligns with the results obtained at 24 d in FGL, where a higher degree of hydrolysis was observed.

Anigbogu et al. [44] examined the effect of an ethanolic extract of Nigerian *J. curcas* leaves on anemic rats. This study observed decreased total cholesterol levels in the groups treated with *J. curcas* extract. Based on these results, the authors report a hypercholesterolemic effect when administering the *J. curcas* ethanolic extract to anemic rats.

On the other hand, the presence of saponins in *J. curcas* L. var. Sevangel cake has been previously reported to have a value of 10.39 HU/mg [1]. Saponins are known to have a hypocholesterolemic effect [45]. This secondary compound in non-toxic *J. curcas* cake may exhibit this bioactivity from the onset of fungal hydrolysis.

Similar results have been found in enzymatic hydrolysis in sunflower seeds with 79% hypocholesterolemic activity in peptide fractions of 1–3 kDa [46], and in peptide fractions of oat protein hydrolysates, 38–85% inhibition of cholesterol micelles [47]. However, in the present study, no enzymatic systems were used, nor was fractionation required to achieve levels close to 80% in the FGL group.

In contrast, a hypocholesterolemic effect has been observed in the soluble fraction of tofu hydrolysates (37.6%) [48], whereas in soy hydrolysates, 48.6% has been reported [39]. These studies show lower percentages than those observed in the FGL group in this study. This is the first report of hypocholesterolemic activity in hydrolysates of *J. curcas* cake.

## 4. Conclusions

The macrofungal hydrolysis systems developed in this study for a non-toxic genotype of *Jatropha* (*J. curcas* L. var. Sevangel) demonstrated in vitro bioactivities including antioxidant, antidiabetic, antihypertensive, and hypocholesterolemic effects. Among these, the system utilizing *G. lucidum* yielded the best results. Previous studies on cake and protein concentrates have highlighted *J. curcas* as a peptide source with antioxidant, antihypertensive, and antidiabetic activities. The generation of bioactive peptides from *J. curcas* cake demonstrated significant nutritional and pharmacological potential for the management of chronic diseases, including cancer, diabetes, hypertension, and dyslipidemia. This is the first report of its cholesterol-lowering activity. Given its multi-bioactive profile, the hydrolysate from *G. lucidum* performed better than the *P. ostreatus* system and was even better than other oilseeds reported in previous studies. These findings are relevant to further studies on the sequencing of peptides with multiple bioactivities, including antioxidant, antidiabetic, antihypertensive, and hypocholesterolemic properties. They also lay the foundation for conducting clinical trials to validate the findings obtained in this in vitro study.

For these reasons, *J. curcas* cake is a by-product emerging as a functional food or nutraceutical ingredient due to its high protein content and its potential to treat diabetes, hypertension, and dyslipidemia and to reduce free radicals. Likewise, low-cost agro-industrial by-products can be used for solid-state fermentation, promoting sustainable practices and contributing to the circular economy.

## Figures and Tables

**Table 1:** The degree of hydrolysis and bioactivities (antioxidant, antidiabetic, antihypertensive, and hypocholesterolemic) of *J. curcas* L var. Sevangel cake by SSF of *P. ostreatus* and *G. lucidum*.

Time (d)	Hydrolysis Degree (ppm)	FRAP (mmol Fe^2+^)	DPPH (mmol Trolox)	DPP-IV Inhibition (%)	ACE-I Inhibition (%)	Hypocholesterolemic Activity (%)
*P. ostreatus*						
0	596.53 ± 8.84 ^d^	24.38 ± 1.16 ^d^	18.11 ± 1.71 ^a^	5.87 ± 0.92 ^d^	51.25 ± 2.03 ^b^	56.48 ± 1.48 ^a^
6	2014.23 ± 26.86 ^a^	24.91 ± 0.41 ^d^	46.17 ± 0.86 ^b^	15.01 ± 1.15 ^c^	61.76 ± 1.22 ^a^	41.55 ± 0.58 ^b^
12	1679.34 ± 44.19 ^c^	35.08 ± 0.52 ^a^	56.17 ± 1.14 ^c^	31.00 ± 1.38 ^b^	65.77 ± 0.25 ^a^	ND
18	2060.85 ± 40.84 ^a^	27.90 ± 1.13 ^c^	57.01 ± 0.34 ^cd^	46.12 ± 1.23 ^a^	11.07 ± 0.98 ^c^	10.17 ± 0.31 ^c^
24	1828.56 ± 22.74 ^b^	31.83 ± 0.77 ^b^	59.70 ± 0.30 ^d^	42.41 ± 1.85 ^a^	ND	46.41 ± 2.99 ^b^
*G. lucidum*						
0	738.97 ± 19.14 ^c^	104.93 ± 0.58 ^a^	10.35 ± 0.90 ^a^	9.79 ± 0.23 ^d^	59.68 ± 1.27 ^b^	69.57 ± 0.10 ^b^
6	1583.77 ± 34.98 ^b^	61.88 ± 4.00 ^c^	16.72 ± 0.95 ^b^	10.44 ± 0.46 ^d^	66.49 ± 1.77 ^a^	50.64 ± 0.72 ^c^
12	1912.67 ± 72.70 ^a^	36.65 ± 0.14 ^d^	22.95 ± 0.37 ^c^	40.95 ± 1.15 ^c^	51.79 ± 0.76 ^c^	50.45 ± 2.17 ^c^
18	1960.59 ± 36.46 ^a^	67.80 ± 1.76 ^b^	26.64 ± 0.52 ^d^	51.88 ± 1.38 ^b^	43.73 ± 0.51 ^d^	72.12 ± 2.55 ^b^
24	2026.47 ± 24.64 ^a^	106.87 ± 0.70 ^a^	24.70 ± 1.00 ^cd^	86.43 ± 0.73 ^a^	10.55 ± 0.73 ^e^	78.13 ± 0.45 ^a^

FRAP: Ferric Reducing Antioxidant Power; DPPH: 2,2-diphenyl-1-picrylhydrazyl; ACE-I: Angiotensin I-converting enzyme; DPP-IV: Dipeptidyl peptidase IV, ND: Not detected. The DPPH and FRAP results are expressed on a per-100 mL hydrolysate basis. All results are expressed as mean ± standard deviation. Different letters across rows of each fungus in the SSF system indicate significant differences (*p* ≤ 0.05). The table shows that the maximum values obtained for the bioactivities of FPO were between 12 and 18 d of hydrolysis, except for hypocholesterolemic activity (0 d). In the case of FGL, the maximum bioactivity values corresponded to 24 d of hydrolysis, except in the case of ACE-I inhibition (6 d).

## Data Availability

The original contributions presented in this study are included in the article/Supplementary Materials. Further inquiries can be directed to the corresponding authors.

## Supplementary Materials

The following supporting information can be downloaded at: https://www.mdpi.com/article/10.3390/foods15020386/s1.

## Abbreviations

The following abbreviations are used in this manuscript:

ACE	Angiotensin I-Converting Enzyme
AOAC	Association of Official Analytical Collaboration
BPs	Bioactive Peptides
DPPH	2,2-diphenyl-1-picrylhydrazyl
DPP-IV	Dipeptidyl peptidase IV
FGL	SSF hydrolyses by Ganoderma lucidum
FPO	SSF hydrolyses by Pleurotus ostreatus
FRAP	Ferric Reducing Antioxidant Power
SSF	Solid-State Fermentation
TNBS	2,4,6-trinitrobenzene sulfonic acid
