# Supplementary material for: Solid-State Fermentation of Jatropha curcas Cake by Pleurotus ostreatus or Ganoderma lucidum Mycelium to Determine Multi-Bioactivities
Source: Foods. 2026 Jan 21;15(2):386. doi: 10.3390/foods15020386 (PMC12841427; doi:10.3390/foods15020386)
Supplement: Supplementary file 1 [file foods-15-00386-s001.zip › 145_01.10.25 (1).pdf]

Image Report: 145_01.10.25 (1)

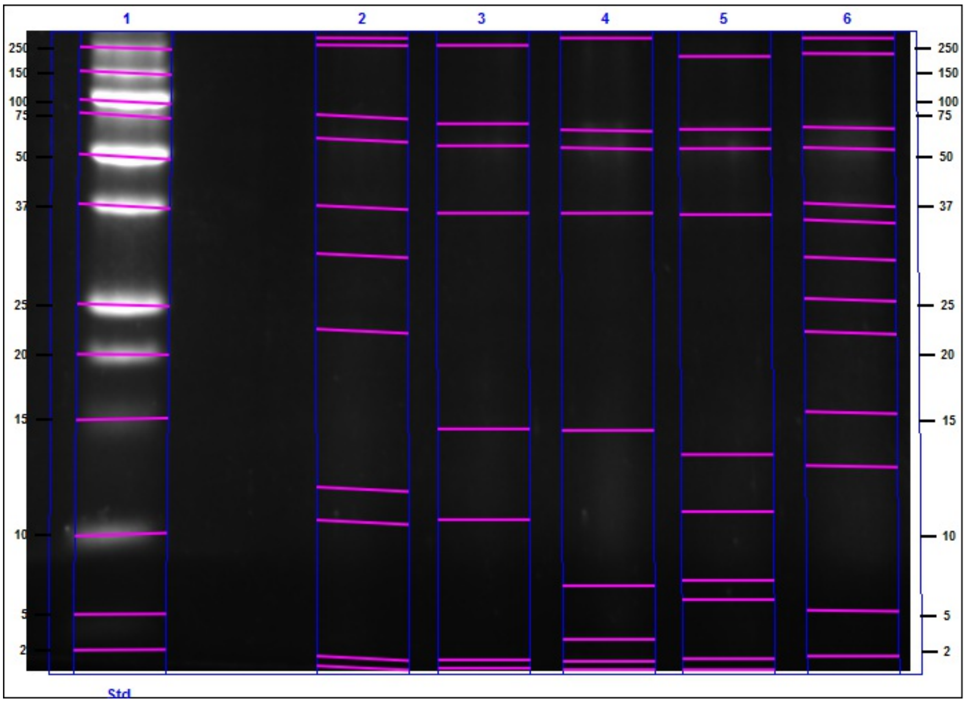

C:\Users\Lab Biotecnologia\Documents\Emmanuel\145_01.10.25 (1).scn

Acquisition Information

Imager	Gel Doc™ EZ
Exposure Time (sec)	0.275 (Auto - Intense Bands)
Application	Coomassie Blue
Dark Type	Referenced
Ref. Bkgd. Time (sec)	10
Flat Field	Applied
Serial Number	735BR05294
Software Version	5.2.1
Illumination Mode	White Transillumination

Image Information

Acquisition Date	02/10/2025 18:39:24
User Name	Lab Biotecnologia
Image Area (mm)	X: 69.3 Y: 50.2
Pixel Size (um)	X: 107.8 Y: 107.8
Data Range (Int)	2185 - 3508

Analysis Settings

Detection	Lane detection:<br>Manually created lanes<br><br>Band detection:<br>Automatically detected bands with sensitivity: High<br>Manually adjusted bands<br><br>Lane Background Subtraction:<br>Lane background subtracted with disk size: 10<br><br>Lane width: 7.22 mm

Mol. Weight Analysis	Standard: precision plus protein dual xtra<br>Standard lanes: first<br>Regression method: Point to Point (semi-log)

Lane And Band Analysis

Lane 1 - precision plus protein dual xtra

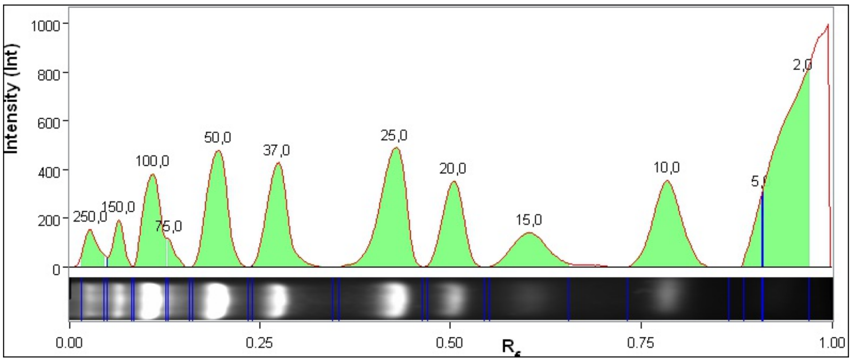

Band No.	Band Label	Mol. Wt. (KDa)	Relative Front	Volume (Int)	Abs. Quant.	Rel. Quant.	Band %	Lane %
1	péptido 1	250,0	0,028	113.297	N/A	N/A	2,3	2,0
2	péptido 2	150,0	0,066	113.230	N/A	N/A	2,3	1,9
3	péptido 3	100,0	0,111	351.281	N/A	N/A	7,1	6,0
4		75,0	0,132	49.580	N/A	N/A	1,0	0,9
5	péptido 4	50,0	0,197	526.486	N/A	N/A	10,6	9,1
6	péptido 5	37,0	0,274	471.546	N/A	N/A	9,5	8,1
7	péptido 6	25,0	0,427	678.107	N/A	N/A	13,7	11,7
8	péptido 7	20,0	0,504	397.846	N/A	N/A	8,0	6,8
9	péptido 8	15,0	0,605	266.459	N/A	N/A	5,4	4,6
10	péptido 9	10,0	0,784	544.777	N/A	N/A	11,0	9,4
11		5,0	0,908	165.289	N/A	N/A	3,3	2,8
12		2,0	0,964	1.271.861	N/A	N/A	25,7	21,9

Band Detection	Automatically detected bands with sensitivity: High
Lane Background	Lane background subtracted with disk size: 10
Lane Width	7.22 mm
Regression Equation	A single equation is not available for this method

Lane 2

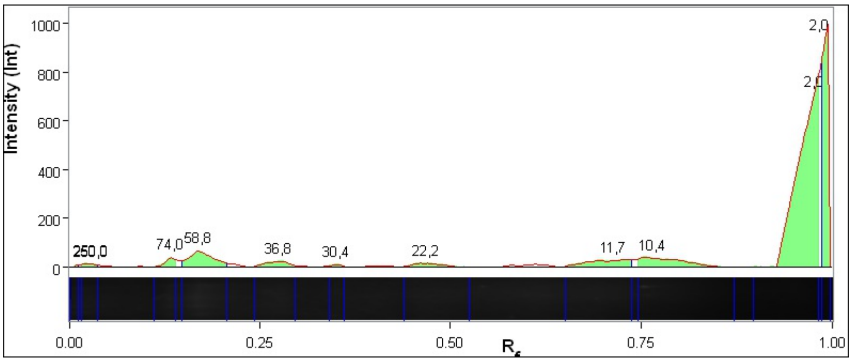

Band No.	Band Label	Mol. Wt. (KDa)	Relative Front	Volume (Int)	Abs. Quant.	Rel. Quant.	Band %	Lane %
1		250,0	0,013	1.675	N/A	N/A	0,1	0,1
2		250,0	0,024	9.380	N/A	N/A	0,6	0,6
3		74,0	0,135	20.837	N/A	N/A	1,4	1,3
4		58,8	0,171	85.358	N/A	N/A	5,7	5,3
5		36,8	0,276	30.083	N/A	N/A	2,0	1,9

6		30,4	0,350	5.293	N/A	N/A	0,4	0,3
7		22,2	0,468	23.986	N/A	N/A	1,6	1,5
8		11,7	0,714	66.665	N/A	N/A	4,4	4,1
9		10,4	0,765	89.847	N/A	N/A	6,0	5,5
10		2,0	0,976	821.018	N/A	N/A	54,5	50,6
11		2,0	0,991	350.946	N/A	N/A	23,3	21,6

Band Detection	Automatically detected bands with sensitivity: High
Lane Background	Lane background subtracted with disk size: 10
Lane Width	7.22 mm
Regression Equation	A single equation is not available for this method

Lane 3

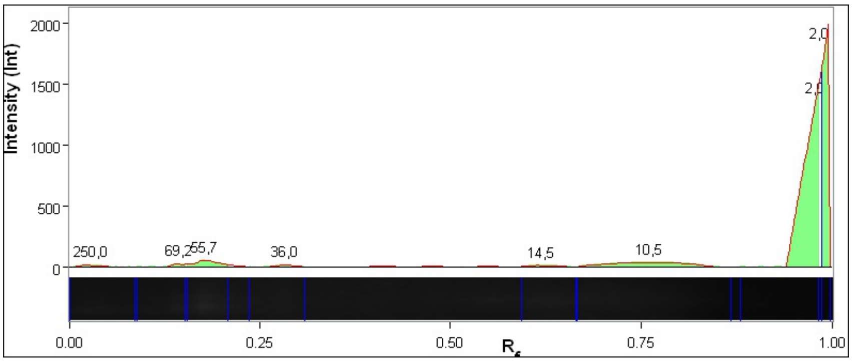

Band No.	Band Label	Mol. Wt. (KDa)	Relative Front	Volume (Int)	Abs. Quant.	Rel. Quant.	Band %	Lane %
1		250,0	0,024	17.621	N/A	N/A	0,8	0,7
2		69,2	0,145	19.229	N/A	N/A	0,9	0,8
3		55,7	0,179	71.891	N/A	N/A	3,2	3,0
4		36,0	0,284	15.879	N/A	N/A	0,7	0,7
5		14,5	0,620	20.234	N/A	N/A	0,9	0,9
6		10,5	0,761	169.443	N/A	N/A	7,7	7,2
7		2,0	0,979	1.209.752	N/A	N/A	54,6	51,1
8		2,0	0,991	689.832	N/A	N/A	31,2	29,1

Band Detection	Automatically detected bands with sensitivity: High
Lane Background	Lane background subtracted with disk size: 10
Lane Width	7.22 mm
Regression Equation	A single equation is not available for this method

Lane 4

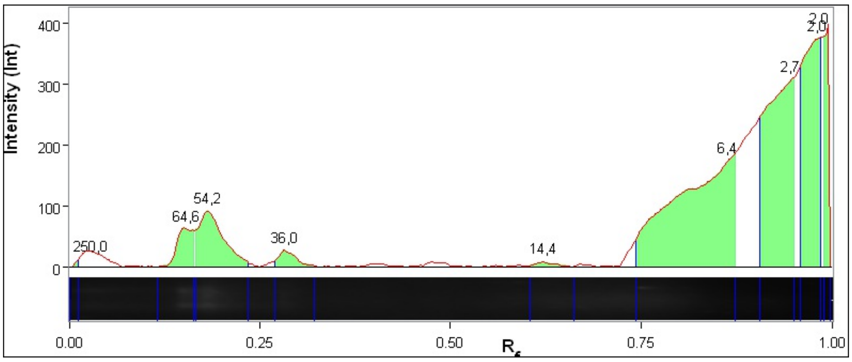

Band No.	Band Label	Mol. Wt. (KDa)	Relative Front	Volume (Int)	Abs. Quant.	Rel. Quant.	Band %	Lane %
1		250,0	0,013	2.412	N/A	N/A	0,1	0,1
2		64,6	0,156	58.156	N/A	N/A	3,3	2,6
3		54,2	0,184	130.248	N/A	N/A	7,3	5,9
4		36,0	0,284	27.135	N/A	N/A	1,5	1,2

5		14,4	0,622	9.581	N/A	N/A	0,5	0,4
6		6,4	0,863	580.488	N/A	N/A	32,7	26,4
7		2,7	0,947	481.395	N/A	N/A	27,1	21,9
8		2,0	0,981	364.145	N/A	N/A	20,5	16,6
9		2,0	0,994	120.064	N/A	N/A	6,8	5,5

Band Detection	Automatically detected bands with sensitivity: High
Lane Background	Lane background subtracted with disk size: 10
Lane Width	7.22 mm
Regression Equation	A single equation is not available for this method

Lane 5

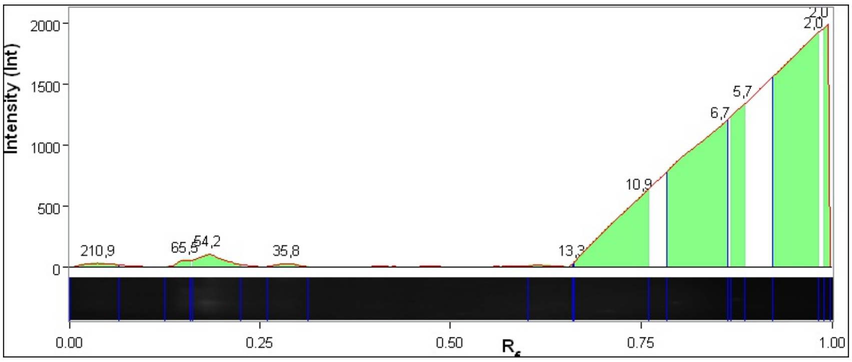

Band No.	Band Label	Mol. Wt. (KDa)	Relative Front	Volume (Int)	Abs. Quant.	Rel. Quant.	Band %	Lane %
1		210,9	0,041	48.106	N/A	N/A	0,5	0,4
2		65,5	0,154	39.999	N/A	N/A	0,4	0,3
3		54,2	0,184	151.353	N/A	N/A	1,5	1,2
4		35,8	0,286	29.815	N/A	N/A	0,3	0,2
5		13,3	0,660	20.569	N/A	N/A	0,2	0,2
6		10,9	0,748	1.255.848	N/A	N/A	12,7	9,9
7		6,7	0,855	2.899.157	N/A	N/A	29,3	22,9
8		5,7	0,885	983.895	N/A	N/A	9,9	7,8
9		2,0	0,976	3.864.292	N/A	N/A	39,1	30,5
10		2,0	0,994	602.397	N/A	N/A	6,1	4,8

Band Detection	Automatically detected bands with sensitivity: High
Lane Background	Lane background subtracted with disk size: 10
Lane Width	7.22 mm
Regression Equation	A single equation is not available for this method

Lane 6

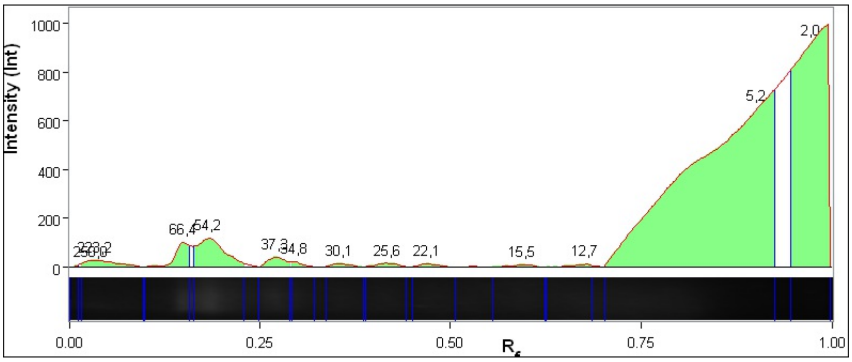

Band No.	Band Label	Mol. Wt. (KDa)	Relative Front	Volume (Int)	Abs. Quant.	Rel. Quant.	Band %	Lane %
1		250,0	0,013	1.608	N/A	N/A	0,0	0,0
2		223,2	0,036	47.570	N/A	N/A	0,9	0,8
3		66,4	0,152	70.819	N/A	N/A	1,4	1,3

4		54,2	0,184	174.669	N/A	N/A	3,4	3,1
5		37,3	0,271	38.525	N/A	N/A	0,8	0,7
6		34,8	0,297	12.060	N/A	N/A	0,2	0,2
7		30,1	0,355	14.070	N/A	N/A	0,3	0,3
8		25,6	0,419	18.425	N/A	N/A	0,4	0,3
9		22,1	0,470	13.601	N/A	N/A	0,3	0,2
10		15,5	0,594	11.122	N/A	N/A	0,2	0,2
11		12,7	0,677	11.390	N/A	N/A	0,2	0,2
12		5,2	0,902	3.033.626	N/A	N/A	59,8	54,0
13		2,0	0,972	1.628.703	N/A	N/A	32,1	29,0

Band Detection	Automatically detected bands with sensitivity: High
Lane Background	Lane background subtracted with disk size: 10
Lane Width	7.22 mm
Regression Equation	A single equation is not available for this method
