# Supplementary material for: Solid-State Fermentation of Jatropha curcas Cake by Pleurotus ostreatus or Ganoderma lucidum Mycelium to Determine Multi-Bioactivities
Source: Foods. 2026 Jan 21;15(2):386. doi: 10.3390/foods15020386 (PMC12841427; doi:10.3390/foods15020386)
Supplement: Supplementary file 1 [file foods-15-00386-s001.zip › 753_30.09.25 (1).pdf]

Image Report: 753_30.09.25 (1)

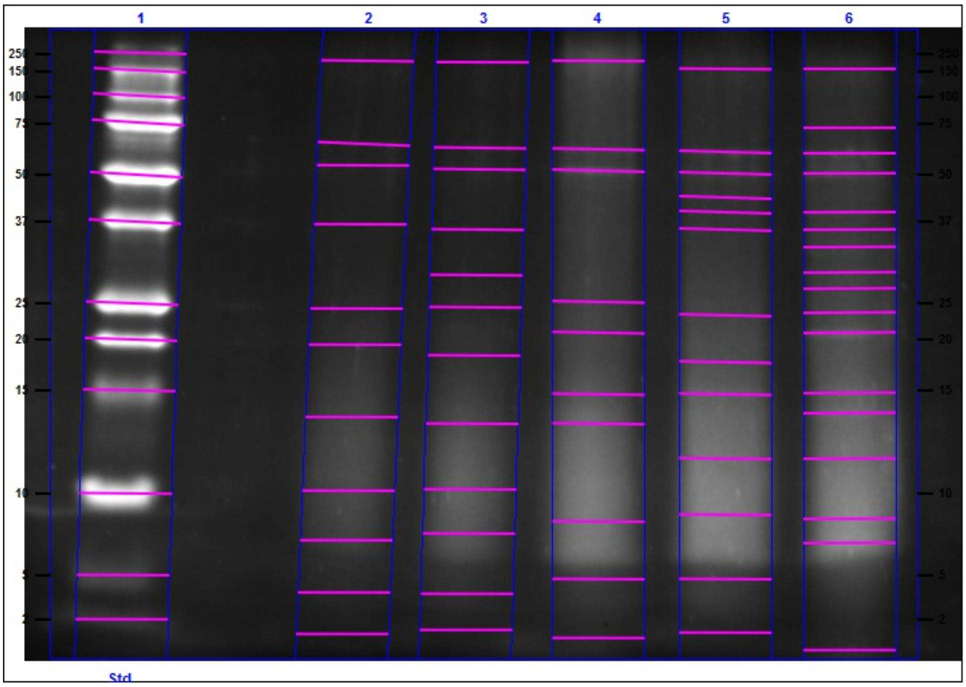

C:\Users\Lab Biotecnologia\Documents\Emmanuel\753_30.09.25 (1).scn

---

Acquisition Information

Imager	Gel Doc™ EZ
Exposure Time (sec)	0.284 (Auto - Intense Bands)
Application	Coomassie Blue
Dark Type	Referenced
Ref. Bkgd. Time (sec)	10
Flat Field	Applied
Serial Number	735BR05294
Software Version	5.2.1
Illumination Mode	White Transillumination

Image Information

Acquisition Date	01/10/2025 18:04:15
User Name	Lab Biotecnologia
Image Area (mm)	X: 75.5 Y: 51.0
Pixel Size (um)	X: 107.8 Y: 107.8
Data Range (Int)	2336 - 3512

Analysis Settings

Detection	Lane detection:<br>Manually created lanes<br><br>Band detection:<br>Automatically detected bands with sensitivity: High<br>Manually adjusted bands<br><br>Lane Background Subtraction:<br>Lane background subtracted with disk size: 10<br><br>Lane width: 7.44 mm

Mol. Weight Analysis	Standard: precision plus protein dual xtra<br>Standard lanes: first<br>Regression method: Point to Point (semi-log)

Lane And Band Analysis

Lane 1 - precision plus protein dual xtra

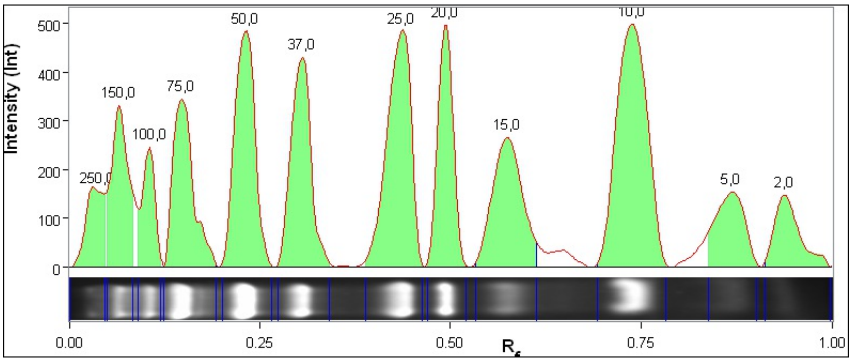

Band No.	Band Label	Mol. Wt. (KDa)	Relative Front	Volume (Int)	Abs. Quant.	Rel. Quant.	Band %	Lane %
1	péptido 1	250,0	0,038	153.042	N/A	N/A	3,4	3,3
2	péptido 2	150,0	0,066	278.415	N/A	N/A	6,3	6,1
3	péptido 3	100,0	0,106	158.769	N/A	N/A	3,6	3,5
4	péptido 4	75,0	0,149	353.832	N/A	N/A	7,9	7,7
5	péptido 5	50,0	0,232	505.287	N/A	N/A	11,3	11,0
6	péptido 6	37,0	0,306	431.112	N/A	N/A	9,7	9,4
7	péptido 7	25,0	0,436	594.918	N/A	N/A	13,4	13,0
8	péptido 8	20,0	0,494	385.986	N/A	N/A	8,7	8,4
9	péptido 9	15,0	0,574	399.372	N/A	N/A	9,0	8,7
10	péptido 10	10,0	0,738	781.218	N/A	N/A	17,5	17,1
11	péptido 11	5,0	0,868	225.147	N/A	N/A	5,1	4,9
12	péptido 12	2,0	0,938	186.783	N/A	N/A	4,2	4,1

Band Detection	Automatically detected bands with sensitivity: High
Lane Background	Lane background subtracted with disk size: 10
Lane Width	7.44 mm
Regression Equation	A single equation is not available for this method

Lane 2

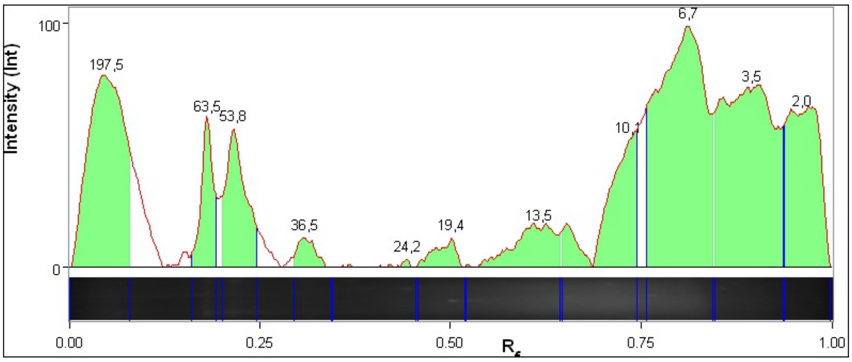

Band No.	Band Label	Mol. Wt. (KDa)	Relative Front	Volume (Int)	Abs. Quant.	Rel. Quant.	Band %	Lane %
1		197,5	0,051	142.071	N/A	N/A	15,5	14,4
2		63,5	0,183	35.880	N/A	N/A	3,9	3,6
3		53,8	0,217	55.200	N/A	N/A	6,0	5,6
4		36,5	0,311	10.626	N/A	N/A	1,2	1,1
5		24,2	0,445	1.518	N/A	N/A	0,2	0,2

6		19,4	0,502	11.799	N/A	N/A	1,3	1,2
7		13,5	0,617	36.639	N/A	N/A	4,0	3,7
8		10,1	0,734	80.109	N/A	N/A	8,7	8,1
9		6,7	0,813	234.876	N/A	N/A	25,6	23,9
10		3,5	0,896	200.859	N/A	N/A	21,9	20,4
11		2,0	0,962	107.502	N/A	N/A	11,7	10,9

Band Detection	Automatically detected bands with sensitivity: High
Lane Background	Lane background subtracted with disk size: 10
Lane Width	7.44 mm
Regression Equation	A single equation is not available for this method

Lane 3

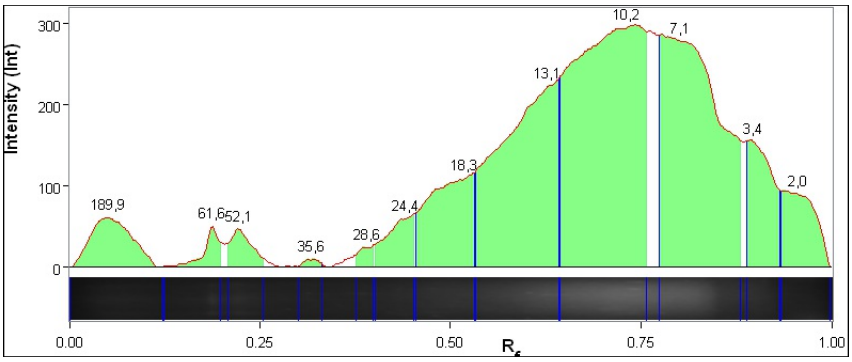

Band No.	Band Label	Mol. Wt. (KDa)	Relative Front	Volume (Int)	Abs. Quant.	Rel. Quant.	Band %	Lane %
1		189,9	0,053	134.964	N/A	N/A	3,7	3,5
2		61,6	0,189	38.640	N/A	N/A	1,1	1,0
3		52,1	0,223	52.578	N/A	N/A	1,4	1,4
4		35,6	0,319	7.797	N/A	N/A	0,2	0,2
5		28,6	0,391	19.803	N/A	N/A	0,5	0,5
6		24,4	0,443	89.148	N/A	N/A	2,4	2,3
7		18,3	0,519	265.374	N/A	N/A	7,3	6,9
8		13,1	0,628	680.064	N/A	N/A	18,6	17,6
9		10,2	0,732	1.106.553	N/A	N/A	30,3	28,7
10		7,1	0,802	890.307	N/A	N/A	24,3	23,1
11		3,4	0,898	206.931	N/A	N/A	5,7	5,4
12		2,0	0,955	164.220	N/A	N/A	4,5	4,3

Band Detection	Automatically detected bands with sensitivity: High
Lane Background	Lane background subtracted with disk size: 10
Lane Width	7.44 mm
Regression Equation	A single equation is not available for this method

Lane 4

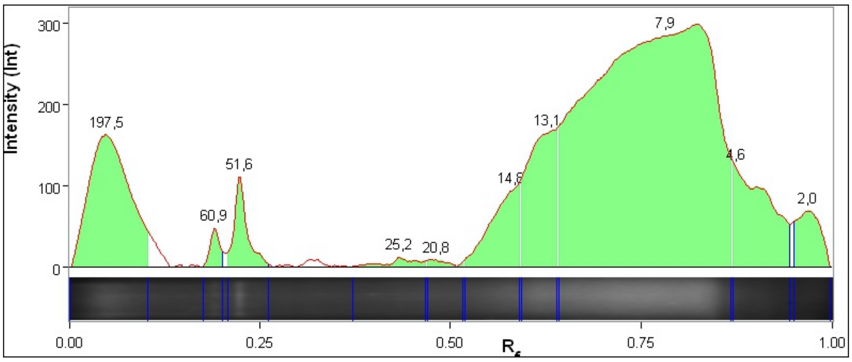

Band No.	Band Label	Mol. Wt. (KDa)	Relative Front	Volume (Int)	Abs. Quant.	Rel. Quant.	Band %	Lane %

1		197,5	0,051	335.961	N/A	N/A	11,1	11,0
2		60,9	0,191	23.253	N/A	N/A	0,8	0,8
3		51,6	0,226	72.657	N/A	N/A	2,4	2,4
4		25,2	0,434	16.974	N/A	N/A	0,6	0,6
5		20,8	0,483	9.315	N/A	N/A	0,3	0,3
6		14,8	0,581	140.415	N/A	N/A	4,7	4,6
7		13,1	0,628	242.880	N/A	N/A	8,1	8,0
8		7,9	0,783	1.860.930	N/A	N/A	61,7	60,9
9		4,6	0,874	227.631	N/A	N/A	7,6	7,5
10		2,0	0,968	83.904	N/A	N/A	2,8	2,7

Band Detection	Automatically detected bands with sensitivity: High
Lane Background	Lane background subtracted with disk size: 10
Lane Width	7.44 mm
Regression Equation	A single equation is not available for this method

Lane 5

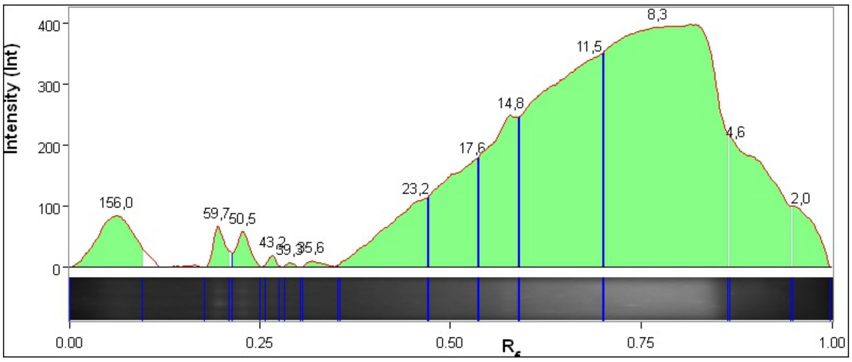

Band No.	Band Label	Mol. Wt. (KDa)	Relative Front	Volume (Int)	Abs. Quant.	Rel. Quant.	Band %	Lane %
1		156,0	0,064	160.287	N/A	N/A	3,2	3,1
2		59,7	0,196	39.330	N/A	N/A	0,8	0,8
3		50,5	0,230	41.745	N/A	N/A	0,8	0,8
4		43,2	0,268	7.590	N/A	N/A	0,1	0,1
5		39,3	0,291	2.553	N/A	N/A	0,1	0,1
6		35,6	0,319	8.142	N/A	N/A	0,2	0,2
7		23,2	0,455	248.124	N/A	N/A	4,9	4,9
8		17,6	0,530	341.343	N/A	N/A	6,7	6,7
9		14,8	0,581	401.718	N/A	N/A	7,9	7,9
10		11,5	0,683	1.167.963	N/A	N/A	23,0	22,9
11		8,3	0,772	2.088.561	N/A	N/A	41,1	40,9
12		4,6	0,874	463.680	N/A	N/A	9,1	9,1
13		2,0	0,960	116.334	N/A	N/A	2,3	2,3

Band Detection	Automatically detected bands with sensitivity: High
Lane Background	Lane background subtracted with disk size: 10
Lane Width	7.44 mm
Regression Equation	A single equation is not available for this method

Lane 6

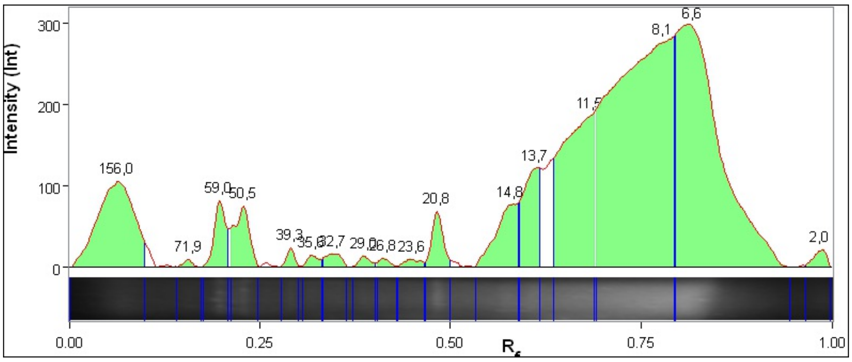

Band No.	Band Label	Mol. Wt. (KDa)	Relative Front	Volume (Int)	Abs. Quant.	Rel. Quant.	Band %	Lane %
1		156,0	0,064	198.444	N/A	N/A	8,1	7,8
2		71,9	0,157	3.864	N/A	N/A	0,2	0,2
3		59,0	0,198	46.437	N/A	N/A	1,9	1,8
4		50,5	0,230	58.512	N/A	N/A	2,4	2,3
5		39,3	0,291	9.591	N/A	N/A	0,4	0,4
6		35,6	0,319	8.901	N/A	N/A	0,4	0,4
7		32,7	0,347	13.731	N/A	N/A	0,6	0,5
8		29,0	0,387	7.935	N/A	N/A	0,3	0,3
9		26,8	0,413	6.141	N/A	N/A	0,3	0,2
10		23,6	0,451	8.073	N/A	N/A	0,3	0,3
11		20,8	0,483	40.296	N/A	N/A	1,6	1,6
12		14,8	0,579	87.975	N/A	N/A	3,6	3,5
13		13,7	0,611	100.464	N/A	N/A	4,1	4,0
14		11,5	0,683	309.396	N/A	N/A	12,6	12,2
15		8,1	0,779	865.881	N/A	N/A	35,3	34,0
16		6,6	0,817	673.923	N/A	N/A	27,5	26,5
17		2,0	0,987	14.145	N/A	N/A	0,6	0,6

Band Detection	Automatically detected bands with sensitivity: High
Lane Background	Lane background subtracted with disk size: 10
Lane Width	7.44 mm
Regression Equation	A single equation is not available for this method
